# Supplementary material for: Transcriptional Profiling of the Effect of Coleus amboinicus L. Essential Oil against Salmonella Typhimurium Biofilm Formation
Source: Antibiotics (Basel). 2023 Nov 7;12(11):1598. doi: 10.3390/antibiotics12111598 (PMC10668763; doi:10.3390/antibiotics12111598)

Figure S1

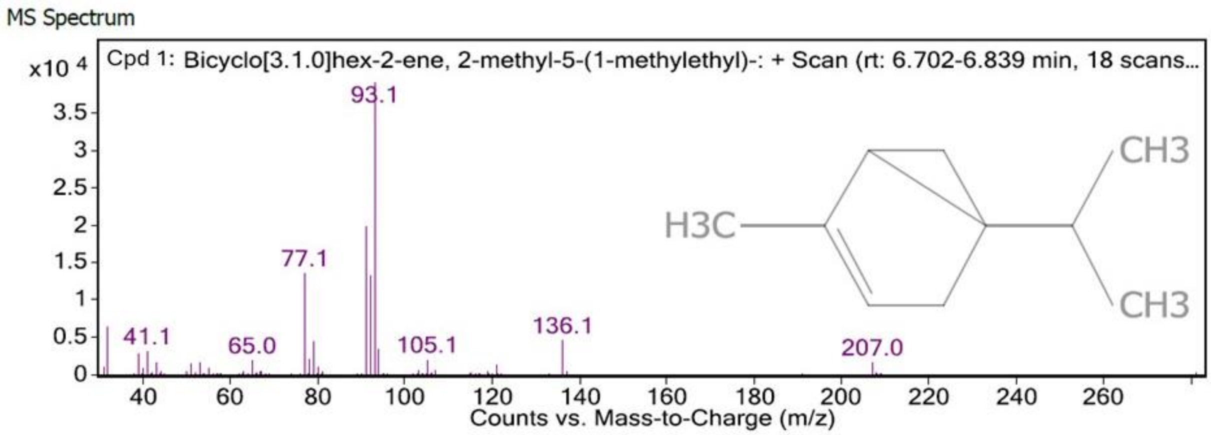
MS Spectrum
x10 4
Cpd 1: Bicyclo[3.1.0]hex-2-ene, 2-methyl-5-(1-methylethyl)-: + Scan (rt: 6.702-6.839 min, 18 scans...
93.1
77.1
41.1
65.0
105.1
136.1
207.0
H3C
CH3
CH3
Counts vs. Mass-to-Charge (m/z)

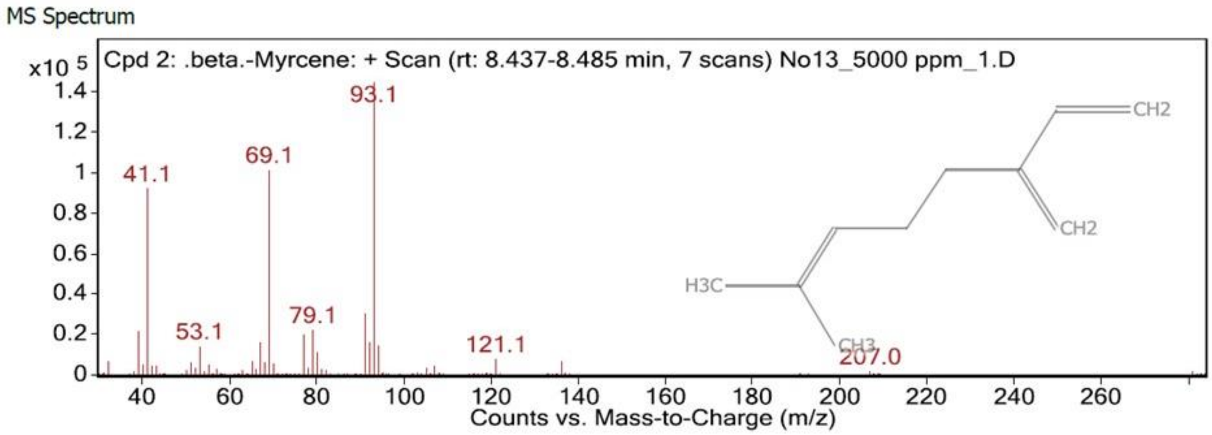
MS Spectrum
x10 5
Cpd 2: .beta.-Myrcene: + Scan (rt: 8.437-8.485 min, 7 scans) No13_5000 ppm_1.D
93.1
69.1
41.1
79.1
53.1
121.1
207.0
CH2
CH2
H3C
CH3
Counts vs. Mass-to-Charge (m/z)

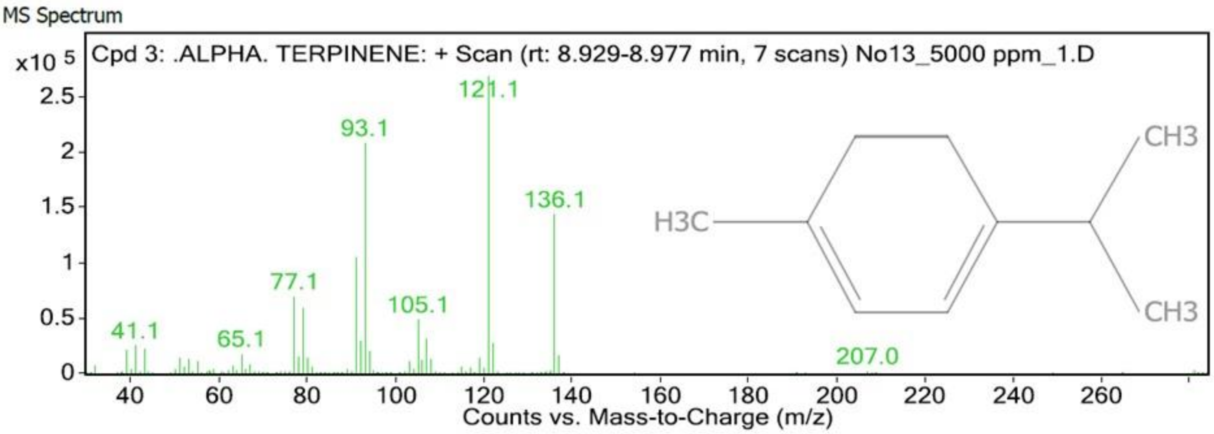
MS Spectrum
x10 5
Cpd 3: .ALPHA. TERPINENE: + Scan (rt: 8.929-8.977 min, 7 scans) No13_5000 ppm_1.D
121.1
93.1
136.1
77.1
105.1
41.1
65.1
207.0
H3C
CH3
CH3
Counts vs. Mass-to-Charge (m/z)

MS Spectrum

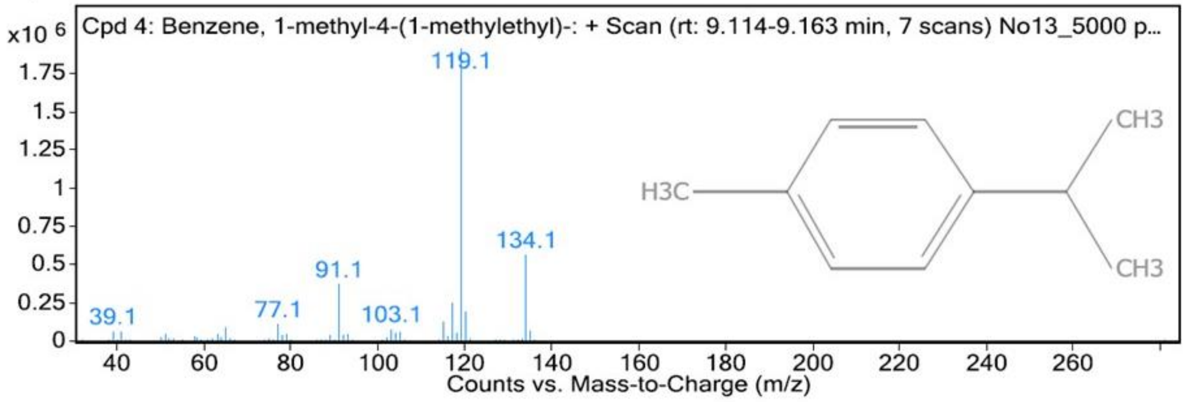

MS Spectrum

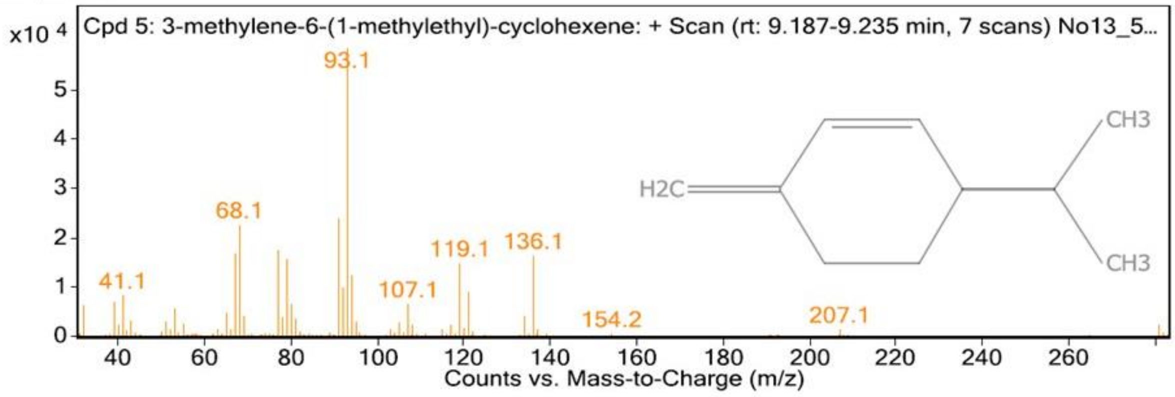

MS Spectrum

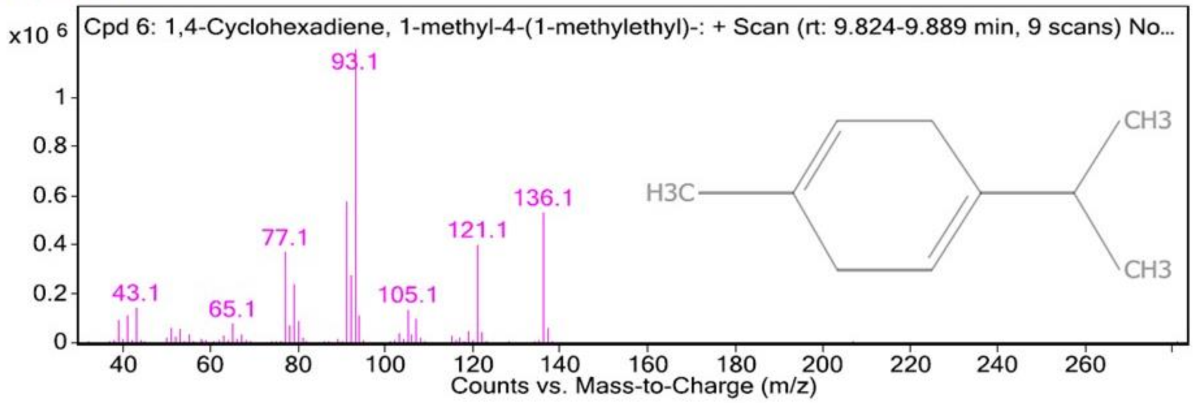

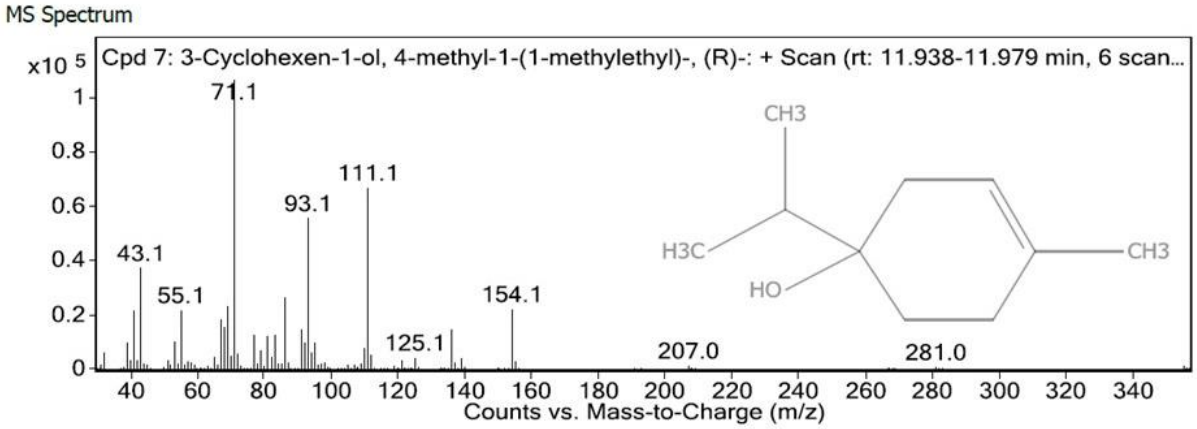
MS Spectrum
x10 5
Cpd 7: 3-Cyclohexen-1-ol, 4-methyl-1-(1-methylethyl)-, (R)-: + Scan (rt: 11.938-11.979 min, 6 scan...
71.1
111.1
93.1
43.1
55.1
154.1
125.1
207.0
281.0
CH3
H3C
HO
CH3
Counts vs. Mass-to-Charge (m/z)

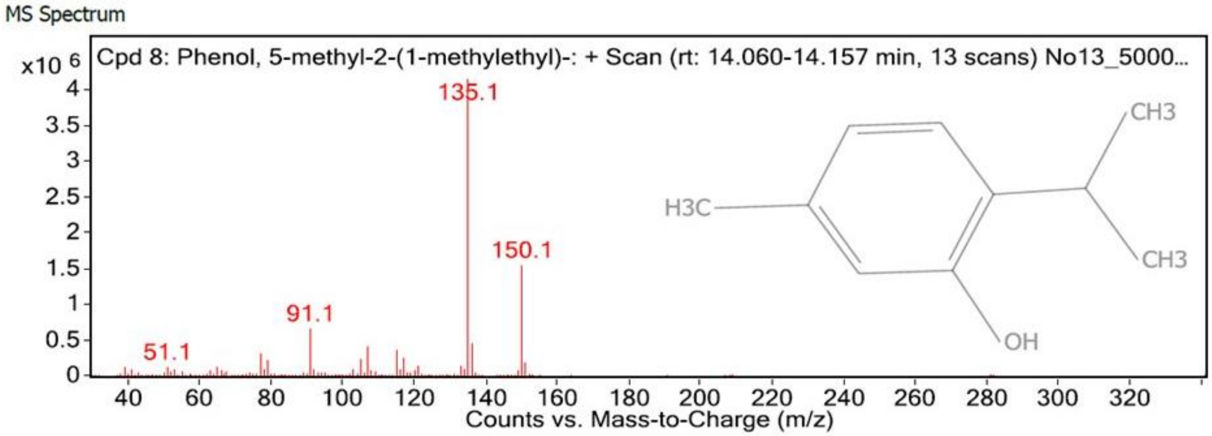
MS Spectrum
x10 6
Cpd 8: Phenol, 5-methyl-2-(1-methylethyl)-: + Scan (rt: 14.060-14.157 min, 13 scans) No13_5000...
135.1
150.1
91.1
51.1
CH3
H3C
CH3
OH
Counts vs. Mass-to-Charge (m/z)

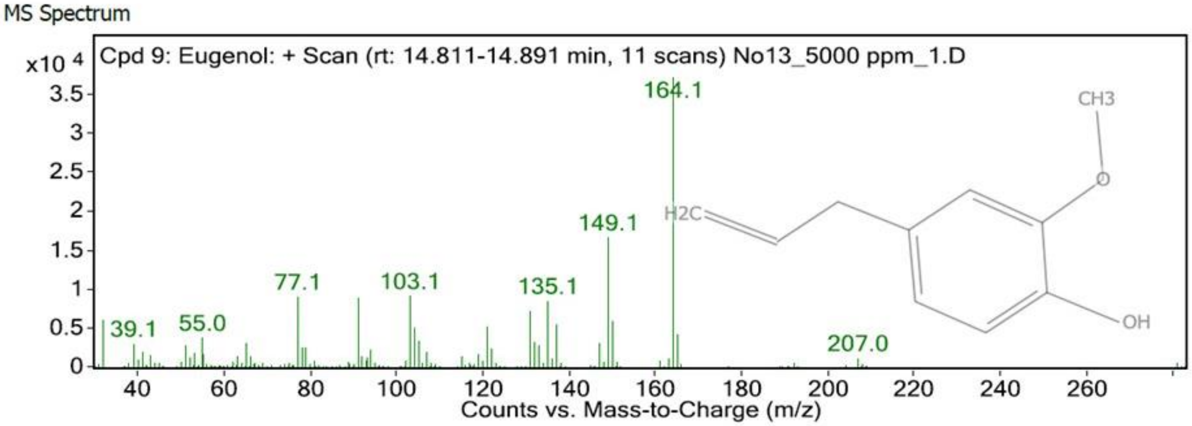
MS Spectrum
x10 4
Cpd 9: Eugenol: + Scan (rt: 14.811-14.891 min, 11 scans) No13_5000 ppm_1.D
164.1
149.1
77.1
103.1
135.1
39.1
55.0
207.0
CH3
O
H2C
OH
Counts vs. Mass-to-Charge (m/z)

MS Spectrum

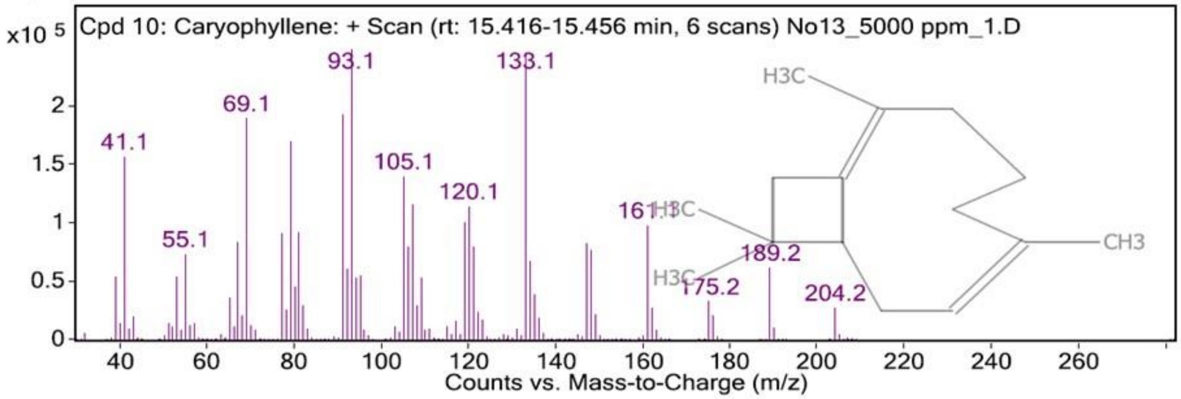

MS Spectrum

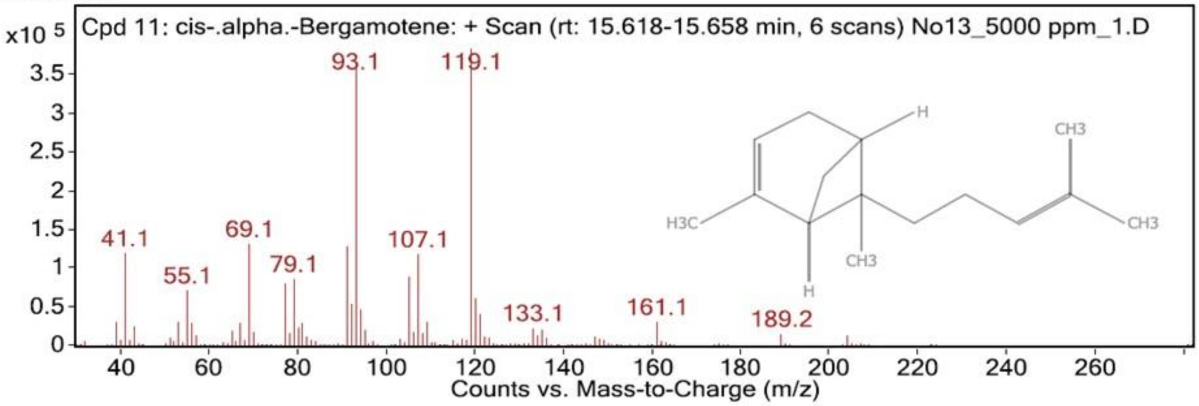

MS Spectrum

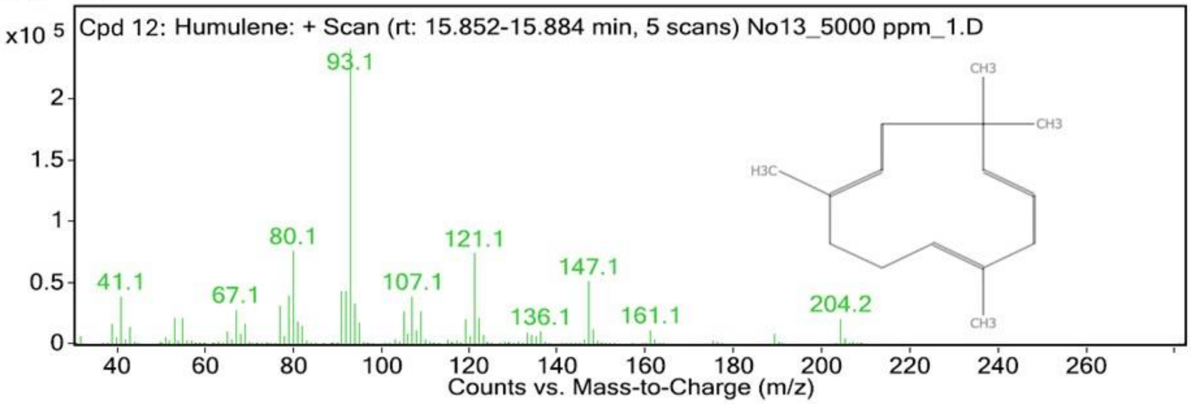

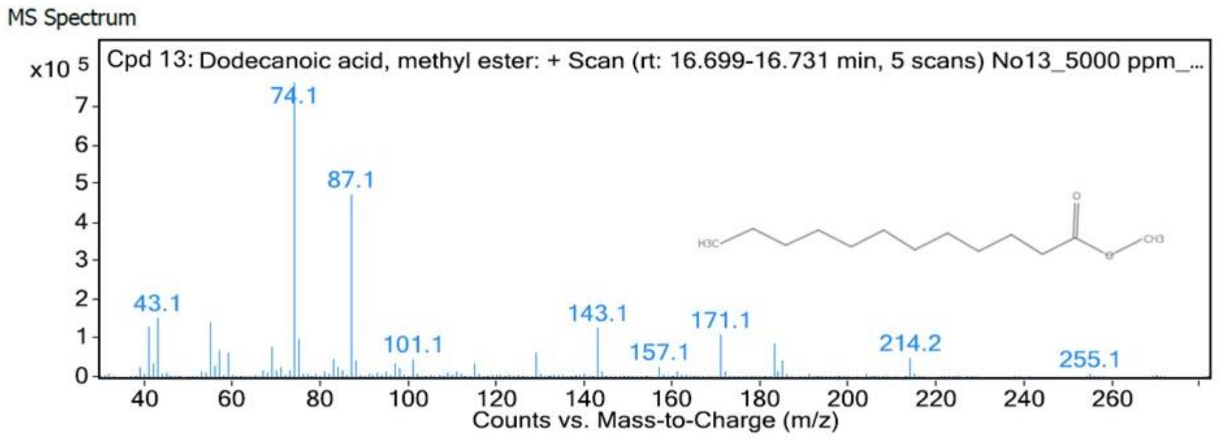
MS Spectrum
x10 5
Cpd 13: Dodecanoic acid, methyl ester: + Scan (rt: 16.699-16.731 min, 5 scans) No13_5000 ppm_...
74.1
87.1
43.1
101.1
143.1
157.1
171.1
214.2
255.1
H3C
CH3
O
Counts vs. Mass-to-Charge (m/z)

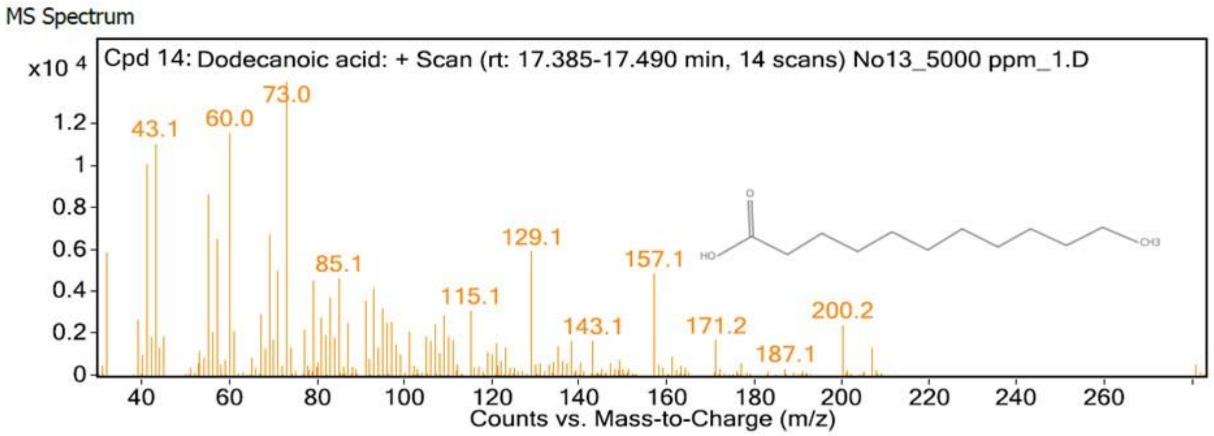
MS Spectrum
x10 4
Cpd 14: Dodecanoic acid: + Scan (rt: 17.385-17.490 min, 14 scans) No13_5000 ppm_1.D
73.0
43.1
60.0
85.1
115.1
129.1
143.1
157.1
171.2
187.1
200.2
HO
CH3
O
Counts vs. Mass-to-Charge (m/z)

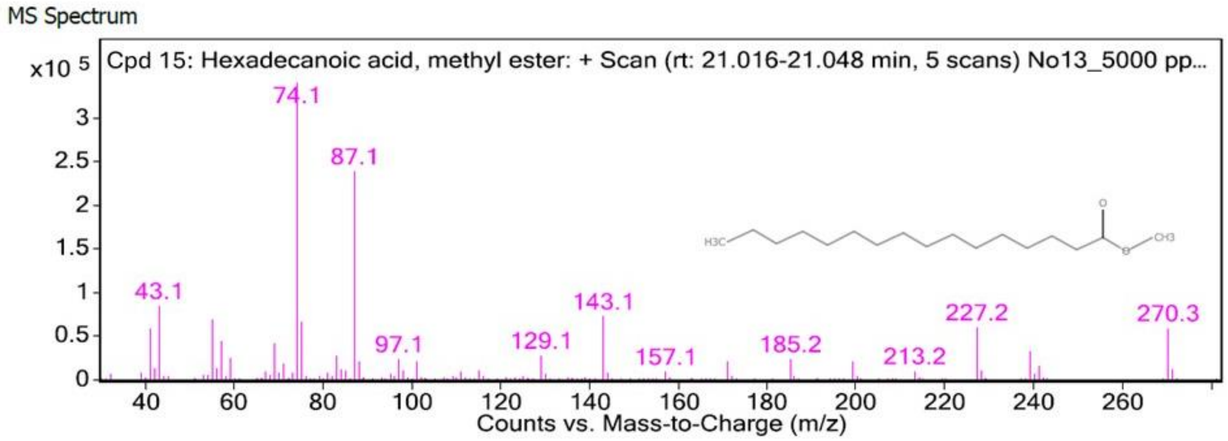
MS Spectrum
x10 5
Cpd 15: Hexadecanoic acid, methyl ester: + Scan (rt: 21.016-21.048 min, 5 scans) No13_5000 pp...
74.1
87.1
43.1
97.1
129.1
143.1
157.1
185.2
213.2
227.2
270.3
H3C
CH3
O
Counts vs. Mass-to-Charge (m/z)

MS Spectrum

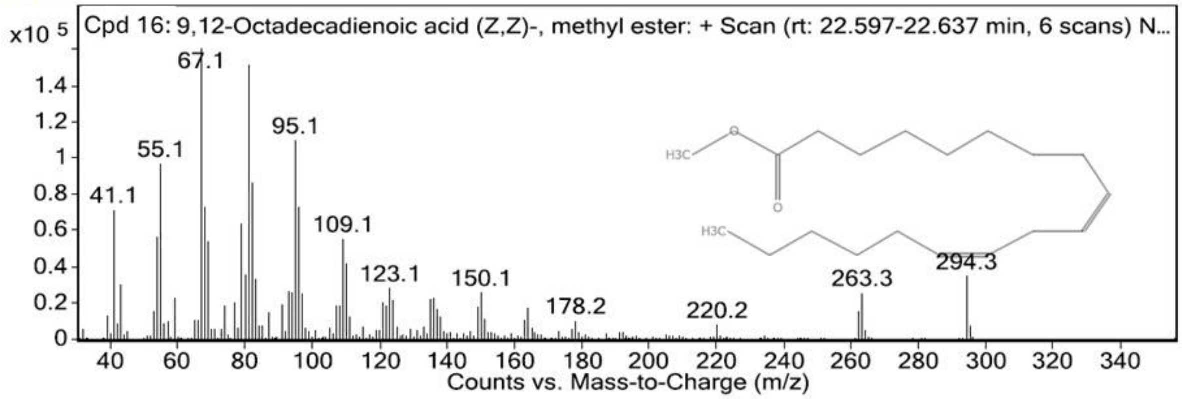

MS Spectrum

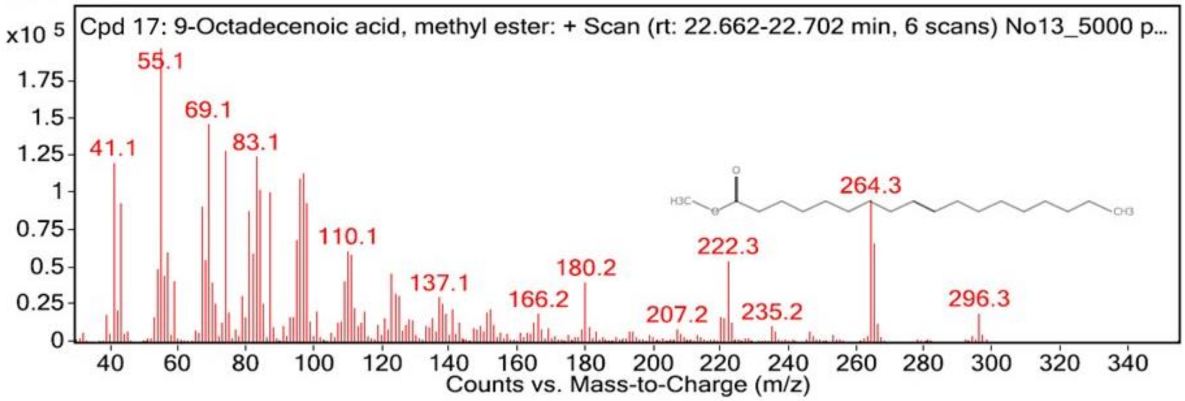

Supplement: Supplementary file 1 [file antibiotics-12-01598-s001.zip › antibiotics-2689683-supplementary.pdf]
